# Processing speed is affected by early impairment in kidney function in the general elder population

**DOI:** 10.1186/s12882-021-02517-5

**Published:** 2021-09-21

**Authors:** Tomas Månsson, Sölve Elmståhl

**Affiliations:** 1 Department of Clinical Sciences in Malmö, Lund University, Jan Waldenströms gata 35, 205 02 Malmö, Sweden; 2 Division of Geriatric Medicine, Skåne University Hospital, Jan Waldenströms gata 35, 205 02 Malmö, Sweden

**Keywords:** Kidney, Renal, Function, GFR, Impairment, Decline, Dementia, MCI, Cognition, Domains

## Abstract

**Background:**

Chronic kidney disease, cardiovascular disease, and cognitive dysfunction are common in the elder population. There is evidence of a connection between these conditions, possibly by a shared vascular pathogenesis. Processing speed is commonly impaired in cerebrovascular disease.

**Methods:**

The data was obtained from the population based study “Good aging in Skåne” (GÅS), and included 905 individuals (mean age = 68 years). We investigated the impact of impaired kidney function at baseline on the development of dementia, MCI, and impairment in specific cognitive domains at follow up 6 years later, using logistic regression models. Impaired kidney function was defined as GFR < 60 ml/min/1,73 m^2^. GFR was estimated from creatinine and cystatin C, using the CKD-EPI formula. Function in the cognitive domains learning and memory, language, complex attention, executive function, perceptual-motor, as well as meta-memory, and global cognitive function, was assessed using a neuropsychological test battery consisting of 12 tests. We compared the test results from follow up, with the results obtained at baseline, using linear regression models in order to assess changes in performance in cognitive domains.

**Results:**

At follow up, 14 and 158 participants had developed dementia and MCI, respectively. We did not find evidence that moderately impaired eGFR at baseline increased the odds of dementia or MCI. A decline in processing speed was associated with impaired kidney function.

**Conclusions:**

The effect on processing speed could represent early vascular implications on cognition. Even at moderately impaired kidney function, overview of cardiovascular risk factors could potentially prevent further cognitive impairment.

## Background

Chronic kidney disease (CKD), cardiovascular disease (CVD), and cognitive decline are common in the elder population [1–3], and constitute major causes behind morbidity and mortality [4–6]. CKD is commonly defined as glomerular filtration rate (GFR) < 60 ml/min/1,73 m^2^ [7, 8].

Previous longitudinal studies investigating GFR as a predictor to cognitive impairment have found inconclusive results, even though studies showing evidence of such an association are in majority [9]. Cognitive impairment is typically studied by assessing the cognitive domains learning and memory, language, complex attention, executive function, perceptual-motor, and social cognition, as described in DSM-5 [10]. Earlier studies investigating which cognitive domains could be associated with impaired kidney function, have found an association with multiple cognitive domains [11], although executive function seems to be more strongly related to kidney function than other domains [9, 12]. We are not aware of studies showing an association between kidney function and meta-memory [13].

The mechanism behind the potential association between impaired kidney function and cognitive function is not fully understood. Both the kidney and the brain are high energy consuming organs, heavily dependent on sufficient blood supply [14]. The kidney and the brain share similar low vascular resistance systems, resulting in microvascular vulnerability to hypertension in these organs [15]. Risk factors of CVD, including hypertension and diabetes, are strongly associated with both CKD [7], and cognitive dysfunction [16, 17]. Cerebrovascular disease is associated with cognitive dysfunction [18], and has been linked to kidney dysfunction [19]. Vascular dementia seems to be overrepresented in people with CKD [20]. The above suggests a possible vascular component in the pathogenesis behind the relationship between CKD and cognitive dysfunction. Cerebrovascular disease tends to affect different areas of the brain, resulting in impairment in multiple cognitive domains. The speed of processing and executive function however, seem to be more affected by cerebrovascular disease than other cognitive domains [18, 21, 22].

We aim to study the impact of renal impairment, defined as eGFR < 60 ml/min/1,73 m^2^, on cognitive development. We define cognitive decline as development of dementia or mild cognitive impairment (MCI) during a 6-year period. We also investigate whether specific cognitive domains are affected by renal impairment.

## Methods

### Study population

The study sample was obtained from the general population cohort study “Good Aging in Skåne” (GÅS), conducted at the Department of Geriatric Medicine, Skåne University Hospital, Sweden. The GÅS study is part of the “Swedish National study on Aging and Care” (SNAC) [23].

Only a brief description of the study is given since the study design is presented elsewhere [24]. Citizens aged 60 and above were randomly selected from the population registry for invitation to participate in the GÅS study. Participants were invited by letter, and to increase the participation rate in fragile subjects, home visits were offered to those unable to attend the research center. The study visit included self-reported questionnaires, collection of blood samples, interview, neuropsychological testing, anthropometrics, physical examination, and medical history. Information of diseases was based on medical records, medical history, information from the national patient registry, and examination by a physician. Participants who attended the first visit are invited to follow up examinations at regular intervals until death.

**Fig. 1:**
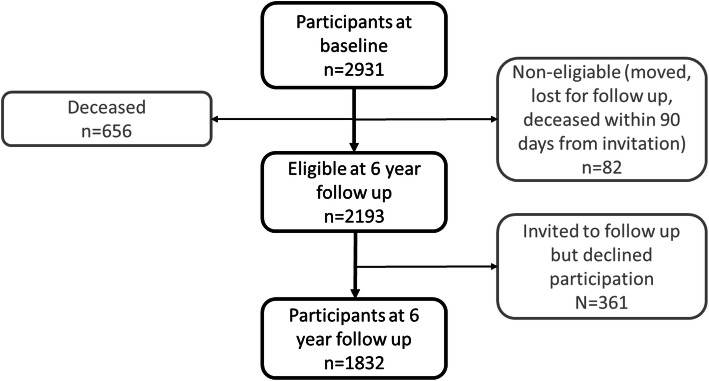
Flow chart of the GÅS participants from baseline to the 6 year follow up. Abbreviations: GÅS = Good Aging in Skåne

**Fig. 2:**
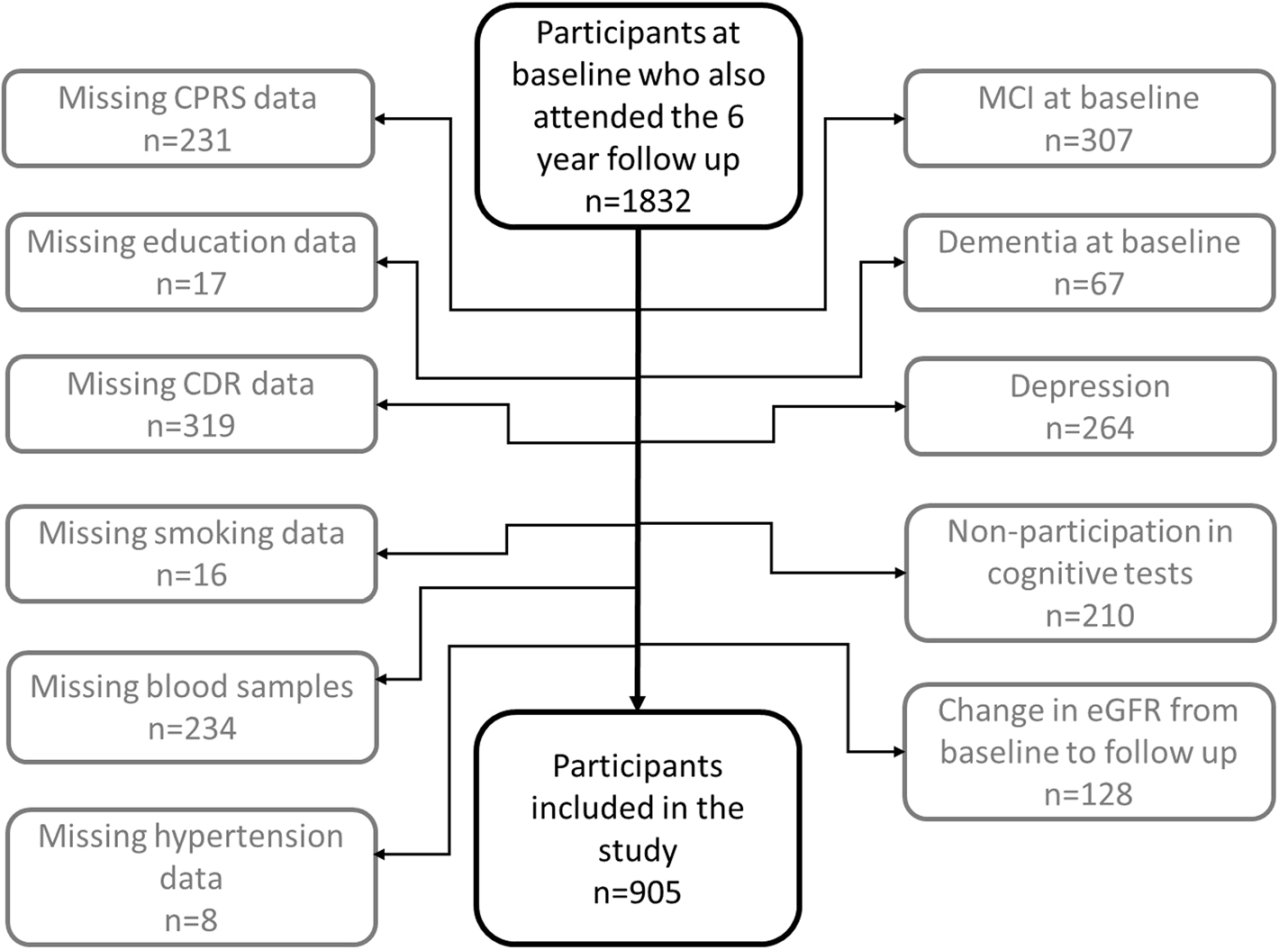
Flow chart of the study sample. The following exclusion criteria were applied both at baseline and at the 6 year follow up: Missing data from CPRS, education, CDR, smoking, and hypertension, as well as missing blood samples, depression, and non-participation in cognitive tests. The exclusion criteria MCI and dementia were applied only at baseline. Abbreviations: CDR = Clinical dementia rating, CPRS = Comprehensive psychopathological rating scale, eGFR = estimated glomerular filtration rate, MCI = Mild cognitive impairment

In this work, we analyzed data from participants from the first wave who also attended the 6-year follow up visit. Description of the study sample is presented in Table 1. Drop outs from baseline to follow up are presented in Fig. 1, and excluded individuals are shown in Fig. 2. Briefly, 2931 participants were examined at baseline between 2001 and 2004. At follow up 6 years later, 1832 of the eligible participants attended (participation rate 81 %). Depression is associated with cognitive impairment [25]. In order to investigate the impact of cognitive worsening due to poor renal function, participants suspected to be suffering from depression at baseline or follow up were excluded (*n* = 264). Individuals with dementia (*n* = 67) or who met the criteria for MCI at baseline (*n* = 307), were excluded. Individuals with missing data in the covariates included in the statistical analyses were excluded (*n* = 490), as well as those who had not participated in any cognitive test (*n* = 210). Individuals who developed kidney impairment or went from impaired to normal kidney function from baseline to follow up, were excluded (*n* = 128). Several participants met multiple exclusion criteria. The final sample size was 905 participants, see Fig. 2.

**Table 1:** Characteristics of the study sample, the drop outs and the excluded participants

Variable	Participants included in the study classified by eGFR		All participants included in the study	Individuals not included in the study		
	eGFR unchanged < 60 from baseline to 6 year follow up	eGFR unchanged from >= 60 from baseline to 6 year follow up		Excluded due to transition from eGFR <60 to >=60 from baseline to follow up or vice versa	Lost to follow up	Excluded^a^
Number	196	709	905	128	1099	799
Age (mean)	78.45 (SD 7.95)	64.65 (SD 5.82)	67.64 (SD 8.52)	71.98 (SD 8.15)	78.67 (SD 10.47)	70.29 (SD 9.68)
Sex						
Women	67.9 %	50.8 %	54.5 %	51.6 %	56.4 %	57.2 %
Men	32.1 %	49.2 %	45.5 %	48.4 %	43.6 %	42.8 %
Education						
≤ fulfilled elementary school	62.2 %	43.6 %	47.6 %	63.3 %	61.0 %	54.5 %
fulfilled secondary school	27.0 %	30.3 %	29.6 %	18.8 %	27.1 %	26.9 %
≥ year of higher education	10.7 %	26.1 %	22.8 %	18.0 %	11.9 %	18.7 %
Hypertension	44.4 %	18.8 %	24.3 %	31.3 %	37.1 %	25.9 %
Diabetes	11.7 %	5.5 %	6.9 %	9.4 %	10%	5.5 %
Smoking habits						
Active smoker	11.2 %	21.2 %	19.0 %	12.5 %	15.1 %	17.2 %
Former smoker	34.2 %	40.6 %	39.2 %	44.5 %	37.0 %	36.4 %
Never smoked	54.6 %	38.2 %	41.8 %	43.0 %	48.0 %	46.4 %
eGFR (mean)	48.44 (SD 9.15)	80.90 (SD 11.48)	73.87 (SD 17.33)	66.28 (SD 8.58)	56.72 (SD 20.34)	70.15 (SD 17.76)
eGFR (median)	50.57 (Q1 43.16, Q3 55.95)	79.52 (Q1 72.24 , Q3 88.82)	76.02 (Q1 62.97, Q3 86.32)	64.76 (Q1 60.57, Q3 70.90)	55.31 (Q1 41.92, Q3 71.03)	71.89 (Q1 57.41, Q3 82.96)

Characteristics of the study sample in the relation to eGFR

eGFR in mL/min/1,73 m^2^ estimated from creatinine and cystatin C using the CKD-EPI formula

*Abbreviations*: *eGFR* estimated glomerular filtration rate, *Q* quartile, *SD* standard deviation

^a^Excluded due to dementia or MCI at baseline, depression, non-participation in cognitive tests, missing data from education, smoking, CRD or missing blood samples

### Kidney function

Glomerular filtration rate was estimated using the chronic kidney disease epidemiology collaboration (CKD-EPI) equation [26]. The mean of eGFR_crea_ and eGFR_cyst_ was used for assessment of estimated glomerular filtration rate (eGFR), since it is more accurate than eGFR_crea_ or eGFR_cyst_ separately [27]. Blood samples were taken nonfasted and cryopreserved at both baseline and follow up. Creatinine and cystatin C were both analyzed by hospital laboratory. Creatinine was analyzed using a modified Jaffe method with a Beckman Coulter LX20 traceable to isotope-dilution mass spectrometry (IDMS), and Cystatin C was analyzed using Gentians reagent with a Beckman Coulter LX 20 [28].

### Cognitive assessment

Dementia was assessed by a physician from the medical records or history, or identified clinically according to the dementia criteria in DSM-IV [29]. A review of the participants’ medical records was also made in order to identify a previous diagnosis of dementia. The comprehensive psychopathological rating scale (CPRS) was used to assess depression [30]. Individuals with a score above 20 were suspected of being depressed [31].

A battery of 12 neuropsychological tests, assessing the following cognitive domains described in the DSM-5 [10], was used: learning and memory, language, complex attention, executive function, and perceptual-motor. Global cognitive function and meta-memory were also assessed.

Learning and memory was assessed using the tests digit span forward [32], free recall, and recognition [33]. In digit span forward, the participant was instructed to repeat a series of numbers. The longest correct digit span was used for assessment. During the test free recall, 16 words were presented to the participant, who then had two minutes to recall as many of these words as possible. The number of correctly recalled words were used for assessment. The recognition test followed free recall. In this test, the 16 words from free recall were presented, along with 16 new words. The task was to identify the 16 words from the free recall test. The number of correctly recalled words, minus the number of false words, were used for assessment.

Language was assessed using the word fluency F and A test [32]. In this test, the task was to name as many words as possible that start with the letter F and then A, during one minute for each letter. The mean of the number of correct words for F and A was used for assessment.

Processing speed is a subdomain to the cognitive domain complex attention, and was assessed using the tests digit cancellation [34], pattern comparison [35], and trail making test (TMT) A [32]. In digit cancellation, the participant was presented with rows of random numbers. The task was to draw a line over as many fours as possible during 30 s. The total number of correct lines was used for assessment. In pattern comparison, figures in pairs were presented. The task was to identify if the pairs were identical or not, during 2 × 30 s. The number of correct answers was used for assessment. In TMT A, the task was to draw lines between numbers in an ascending order. The time, in seconds, it took to complete the test was used for assessment.

Executive function was assessed using the test digit span backwards [36], and TMT B [32, 37]. In digit span backwards, the task was to repeat a series of numbers backwards. The longest correct digit span was used for assessment. In TMT B, the task was to draw lines between numbers and letters alternately, in an ascending order. The time, in seconds, it took to complete the test was used for assessment.

The perceptual-motor domain was assessed using a mental rotations test [38, 39]. In this test, the task was to identify rotated versions of 3-dimensional figures. The number of correct answers, divided with the number of answered questions, were used for assessment.

Global cognitive function was assessed using the mini mental state examination (MMSE) test [40].

Meta-memory was assessed using a confidence judgement test [41]. In this test, the first task was to answer questions of general knowledge. The second task was to state how certain (in percent) the participant had been of answering the questions correctly. The confidence was estimated using a confidence calibration formula. 0 means perfect calibration, that is, the ability of the participant to answer the questions correctly or not is exactly what he/she expects. The higher the value from the calibration formula, the more the participant is misjudging his/her ability of answering the questions correctly.

For more details regarding assessment of cognition in this study, we refer to the method section in [13].

Mild cognitive impairment was defined as having a combination of self-reported memory complaint, objective cognitive complaint, preserved independence in functional abilities, and not suffering from dementia [42]. The clinical dementia rating (CDR) scale [43] was used to assess functional abilities. A score of 1 and above in any of the 6 items included in CDR was considered as representative of functional dependence.

Objective cognitive complaint was defined as performing worse than 1.5 standard deviations (SD) from a reference population mean in at least one cognitive test, representing any of the cognitive domains described above [44, 45]. The reference population consisted of 3363 individuals in the same age span, from the study SNAC-Kungsholmen [23]. After exclusion due to dementia, depression, or missing data, 2730 individuals remained. Participants who had a score below 1.5 SD from that of the reference population were considered to have an impairment. Since cognitive function is affected by age, sex and educational level, cut-off values were calculated for different combinations of age, sex and educational level.

In order to study which cognitive domains were affected, MCI was further divided into 4 subgroups [42]: amnestic MCI single domain (aMCIs), amnestic MCI multiple domains (aMCIm), nonamnestic MCI single domain (naMCIs), and nonamnestic MCI multiple domains (naMCIm). The objective complaint in aMCIs was defined as performing worse than 1.5 SD from mean of the reference population in the domain learning and memory, but no other domains. The objective complaint in aMCIm was defined as performing worse than 1.5 SD from the reference population mean in the learning and memory domain, and at least one more domain. The objective complaint in naMCIs was defined as performing worse than 1.5 SD from mean of the reference population in one domain, but not the learning and memory domain. The objective complaint in naMCIm was defined as performing worse than 1.5 SD from mean of the reference population in multiple domains, but not the learning and memory domain.

### Statistical methods

In order to study the effect of poor kidney function on cognition, two groups were identified at baseline based on eGFR level: Those with impaired kidney function, defined as eGFR < 60 ml/min/1,73 m^2^, and those with normal kidney function, defined as eGFR ≥ 60 ml/min/1,73 m^2^. After 6-year follow up, some participants may have improved, worsened, or kept a similar eGFR level. In order to assure that changes in cognition did not occur before changes in kidney function, we focused on patients who remained stable in their kidney function. The outcomes of main interest at 6-year follow up were the occurrence of dementia and MCI in previously cognitive intact participants.

The odds of developing dementia at follow up were calculated using a logistic regression model. A similar approach was implemented to estimate the odds of developing MCI. The odds of developing a particular MCI subtype at follow up were calculated using a multinomial logistic regression model.

To assess change in cognitive function in the different cognitive domains, we compared the results of the cognitive tests at the 6-year follow up visit with the results at the baseline visit. We calculated the difference in result for each cognitive test, and using linear regression models, we investigated whether the mean difference in each test differed between participants with normal and poor kidney function.

Age, sex, level of education, as well as hypertension, diabetes, and smoking habits have the potential to affect cognitive impairment and renal function. Therefore, these variables were controlled for in the statistical models. Level of education was defined as elementary school not completed or fulfilled elementary school, fulfilled secondary school, and one year or more of higher education or university degree. Hypertension was defined as systolic blood pressure ≥ 140 mmHg and/or diastolic blood pressure ≥ 90 mmHg taken by a nurse [46], and/or current treatment for hypertension. Diabetes was assessed in the questionnaire, by asking the participant of a prior diagnosis of type 1 or type 2 diabetes. To avoid misclassification, a review of the participants’ medical records was also made, in order to find a prior diagnosis of hypertension and/or diabetes. Smoking habits were defined as active smoker, former smoker or never smoked.

Sample size calculations were not performed for this study. The width of the confidence intervals is used as an estimate of precision. The statistical significance level was set to 0.05. The objectives of the study were exploratory, and therefore correction for multiple comparisons were not implemented. The statistical analyses described above were performed using the IBM SPSS version 25 software.

## Results

We observed that 196 participants had a remaining eGFR of < 60 mL/min/1,73 m^2^, and 709 a remaining eGFR of ≥ 60 mL/min/1,73 m^2^, from baseline to the follow up visit. 101 participants developed impairment in the renal function, and 27 went from impaired to normal kidney function, from baseline to follow up. After 6 years, 14 participants developed dementia and 158 participants fulfilled the MCI criteria.

We observed that having impaired renal function does not seem to increase the odds of developing dementia over 6 years (odds ratio (OR): 2.33, 95 % confidence interval (CI) (0.48, 11.28), or MCI (OR: 0.64, 95 % CI (0.97, 1.03). In other words, we did not observe an association between impaired kidney function and cognitive decline over a 6-year period, see Table 2. A reversed association between impaired kidney function and the development of naMCIs, but not aMCIs, aMCIm or naMCIm was found.

**Table 2:** Incidence of dementia, MCI and MCI subtypes at follow up based on kidney function at baseline

Outcome	Number of incidents from baseline to follow up (cumulative incidence in % in parenthesis)			OR^a^ (95 % CI for OR) *p*–value
	All participants (*n*=905)	Participants with eGFR < 60 mL/min/1,73 m2 from baseline to follow up (*n*=196)	Participants with eGFR ≥ 60 mL/min/1,73 m2 from baseline to follow up (*n*=709)	All participants (*n*=905)
Dementia	14 (1.5 %)	10 (5.1 %)	4 (0.6 %)	2.33 (0.48–11.28) 0.29
MCI	158 (17.5 %)	26 (13.3 %)	132 (18.6 %)	0.64 (0.35–1.17) 0.15
aMCIs	37 (4.1 %)	7 (3.6 %)	30 (4.2 %)	0.98 (0.32–3.07) 0.98
aMCIm	25 (2.8 %)	7 (3.6 %)	18 (2.5 %)	1.51 (0.43–5.34) 0.53
naMCIs	83 (9.2 %)	10 (5.1 %)	73 (10.3 %)	0.41 (0.17–0.99) 0.05
naMCIm	13 (1.4 %)	2 (1.0%)	11 (1.6 %)	0.34 (0.05–2.43) 0.28

Binary logistic regression models were used to calculate OR for dementia and MCI

A multinomial logistic model was used to calculate OR for the MCI subtypes

All calculations were adjusted for the following covariates: age, sex, level of education, hypertension, diabetes, and smoking habits

*Abbreviations*: *eGFR* estimated glomerular filtration rate, *CI* confidence interval, *OR* odds ratio

^a^Reference level for OR = Participants with eGFR ≥ 60 mL/min/1,73 m2 from baseline to follow up

We also investigated the impact of impaired kidney function on decline in specific cognitive domains, by measuring if a difference in each cognitive test score between the baseline visit and the follow up visit could be associated with renal impairment, (see Table 3). A statistically significant association between impaired kidney function at baseline to follow up, and worse performance on the pattern comparison test, was found. The average change in score for pattern comparison was 1.18 points, 95 % CI (-2.26, -0.09), *p*-value: 0.03. For comparison, baseline data from participants with normal kidney function in this study, shows that the age group 60–64 years performed 2.35 points better than the age group 65–69 years. While, in general, a larger delta score was seen in the cognitive test results from baseline to follow up in the group with impaired kidney function, no other statistically significant difference in delta score between the two eGFR groups was found. A trend between having impaired kidney function at baseline to follow up, and worse performance on the TMT A test at follow up, was observed. This association was not statistically significant however (*p*-value 0.08).

**Table 3:** Performance on the cognitive tests at follow up based on kidney function at baseline

Cognitive test	Cognitive domain	Mean result baseline		Mean result 6 year follow up		Mean change in test result from baseline to 6 year follow up		Mean difference in test result change between baseline and follow up (95 % CI) *p*–value
		Participants with eGFR < 60 mL/min/1,73 m2 from baseline to follow up (n)	Participants with eGFR ≥ 60 mL/min/1,73 m2 from baseline to follow up (n)	Participants with eGFR < 60 mL/min/1,73 m2 from baseline to follow up (n)	Participants with eGFR ≥ 60 mL/min/1,73 m2 from baseline to follow up (n)	Participants with eGFR < 60 mL/min/1,73 m2 from baseline to follow up (n)	Participants with eGFR ≥ 60 mL/min/1,73 m2 from baseline to follow up (n)	
MMSE	Global	26.79 (192)	27.83 (708)	25.66 (184)	27.43 (701)	–1.13	–0.40	–0.11 (–0.61, 0.38) 0.66
Digit span forwards	Learning and memory	5.62 (194)	5.80 (708)	5.27 (195)	5.62 (708)	–0.35	–0.18	–0.04 (–0.28, 0.20) 0.75
Free recall	Learning and memory	6.55 (190)	7.64 (699)	5.67 (193)	7.28 (707)	–0.88	–0.36	0.13 (–0.37, 0.63) 0.62
Recognition	Learning and memory	11.73 (188)	12.43 (695)	10.82 (193)	12.15 (703)	–0.91	–0.28	–0.09 (–0.79, 0.61) 0.81
Word fluency	Language	11.38 (196)	13.56 (706)	10.87 (193)	13.42 (705)	–0.51	–0.14	0.05 (–0.68, 0.78) 0.89
Digit cancellation	Complex attention	15.82 (189)	18.68 (704)	15.06 (170)	18.82 (698)	–0.76	0.14	–0.30 (–0.96, 0.35) 0.36
Pattern comparison	Complex attention	23.61 (189)	30.45 (703)	21.46 (170)	30.03 (697)	–2.15	–0.42	–1.18 (–2.26, –0.09) 0.03
TMT A^a^	Complex attention	16.53 s (183)	12.62 s (682)	18.79 s (165)	12.02 s (674)	2.26 s	–0.60 s	1.23 (–0.17, 2.62) 0.08
TMT B^a^	Executive function	36.33 s (141)	25.37 s (628)	47.66 s (128)	26.23 s (621)	11.33 s	0.86 s	1.19 (–3.90, 6.28) 0.65
Digit span backwards	Executive function	4.02 (194)	4.38 (707)	3.82 (194)	4.26 (708)	–0.20	–0.12	–0.04 (–0.28, 0.21) 0.78
Mental rotations^b^	Perceptual motor	0.57 (181)	0.65 (696)	0.56 (175)	0.61 (702)	–0.01	–0.04	0.02 (–0.03, 0.06) 0.52
Confidence judgement^c^	Meta–memory	0.09 (191)	0.11 (697)	0.09 (194)	0.10 (708)	0.00	–0.01	0.01 (–0.02, 0.03) 0.62

Linear regression models were used to calculate the delta result for each cognitive test from baseline to follow up between the two eGFR groups

All analyses were adjusted for age, sex, level of education, hypertension, diabetes, and smoking habits at baseline

*Abbreviations*: *eGFR* estimated glomerular filtration rate, *CI* confidence interval, *n* number, *s* seconds

^a^The time (in seconds) to complete the TMT A and the TMT B test was measured

^b^In mental rotations, the proportion of correct answers divided with the total number of answered questions was calculated

^c^ A calibration formula was used to calculate confidence. 0 means perfect confidence judgement. The bigger the value, the worse confidence judgement

## Discussion

In this longitudinal study, we present a thorough assessment of the impact of early onset renal impairment on cognitive decline during a 6-year period in an elder population.

The incidence of dementia from baseline to follow up did not differ statistically significantly between the groups based on renal function (*p*-value 0.29). The cumulative incidence of dementia from baseline to follow up was 5.1 % in the group with impaired renal function, compared to only 0.6 % in the group with normal renal function. While this difference is quite large, a possibility is that this difference could be explained by factors other than renal impairment, that we did not consider. The number of incidents of dementia from baseline to follow up was also quite small (*n* = 14). Therefore, the conclusion that impaired kidney function does not precede dementia based on a non-significant p-value seen in this study, can probably not be made.

The cumulative incidence of MCI from baseline to follow up differed less between the groups. 13.3 % developed MCI in the group with impaired renal function from baseline to follow up, compared to 18.6 % in the group with normal renal function. Again, the difference was not statistically significant.

In a study by Overton et al. [47], 5 % of individuals with MCI progressed into dementia in a 6-year follow up. A possible reason a larger proportion of participants with impaired kidney function developed dementia, and conversely, a smaller proportion (compared to the group with normal kidney function) fulfilled the MCI criteria at follow up, could be that a greater proportion of participants in the group with impaired renal function, converted from MCI to dementia during the 6-year follow up time.

The population in this study was relatively young (mean age = 68 years). A mean eGFR of 74 ml/min/1,73 m^2^ suggests that they also were relatively healthy. Even though home visits were offered those unable to attend the research center, individuals with more severe morbidity were most likely underrepresented. Therefore, it is expected that only few participants with dementia are represented in the study, which may partially explain the lack of statistical significance. This is supported by a comparison from the Framingham heart study (*n* = 5205) [48], where the incidence rate of all cause dementia was 40–72 per 10 000 person years (adjusted for age and sex), compared to 26 per 10 000 person years in our study material.

The diagnosis of MCI is not straightforward. The first definition of MCI was elaborated from investigators at the Mayo clinic in the late 1990 s [44]. Initially, since MCI was regarded as a precursor to Alzheimer´s disease (AD), decline in memory was the only cognitive domain considered. The idea of MCI as a precursor to AD came to be challenged, and at a symposium in 2003, the definition of MCI was revised to include decline in any cognitive domain [49]. Multiple workgroups have since worked on a definition of MCI. Consensus seems to exist on the following four criteria: self- or informant-reported cognitive complaint, objective cognitive complaint, preserved independence in functional abilities, and the absence of dementia [42]. In this study, we used the initial Mayo criteria of self-reported memory complaint. Informant cognitive complaint, as well as functional abilities, were assessed using CDR. This may have affected the incidence of MCI in our sample, and also contributed to a higher proportion of individuals with a precursor state to AD in the MCI group. In addition, it is known that MCI can develop into dementia, but it has also been shown that over 50 % of individuals with MCI revert back to normal cognitive functioning over time [47], and therefore a potential misclassification in MCI could be expected.

The number of patients who fulfilled the criteria for the different MCI subtypes was overall low, which hampers the estimation of the OR. Furthermore, we observed that differences in MCI subtypes between the group with impaired, versus the group with non-impaired renal function, also were low. At follow-up, a lower incidence of naMCIs was noted in subjects with impaired kidney function in conjunction with a numerically higher incidence of dementia. This finding might be explained by a worse development of impaired cognition in the subjects with impaired kidney function, also supported by a worse decline in the complex attention test pattern comparison in the same group adjusted for confounders. An alternative explanation might be a false positive finding due to multiple comparisons.

Evidence of a potential association between severe kidney impairment and cognitive decline was presented in a recent longitudinal review study (*n* = 5796) by Zijlstra et al. [50]. The authors found that in older individuals with vascular burden, only severe kidney dysfunction, correspondent to eGFR < 30 ml/min/1,73 m^2^ [7, 8], was associated with cognitive decline over time.

The mean eGFR in the group with impaired kidney function in this study was 48 ml/min/1,73 m^2^, representing a moderately impaired kidney function. The representation of participants with severe decline in kidney function was limited in our sample. Only 8 individuals out of 1033 had an eGFR < 30 ml/min/1,73 m^2^. It remains unclear which degree of kidney impairment would onset a worsening in cognitive function. We speculate that a larger proportion of participants with severe kidney impairment would probably have increased the odds of developing dementia and MCI.

We observed that impaired kidney function preceded worse performance on the pattern comparison test. This test measures processing speed, as part of the cognitive domain complex attention [10, 35]. Processing speed is defined as the time it takes for a person to interpret and respond to a stimulus in the environment [51]. A trend between impaired kidney function at baseline to follow up and worse performance on TMT A, which also measures processing speed [32], was seen, although this association did not reach statistically significance. Longitudinal studies on risk factors of CVD like hypertension, and metabolic-related conditions such as diabetes and obesity, indicate increased risk for deficits in attention and executive function [52]. The same risk factors are also involved in the development of kidney disease [53]. In a large review study (*n* = 89 822), investigating the relationship between CKD and cognitive decline in mostly (but not exclusively) older individuals, Zammit et al. [9] found an association between GFR and executive dysfunction, and to a lesser degree with the speed of processing, global cognition and memory. Our findings, therefore, support a possible vascular component as a significant factor to the relationship between early onset of impaired kidney function and cognition.

The speed of processing, as well as executive function and memory, are fluid cognitive abilities, that is, abilities that are innate and correspond to an individual´s ability to process information and solve problems. Crystallized cognitive abilities on the other hand, like vocabulary and generalized knowledge, refers to abilities that are learned. Fluid cognitive abilities are expected to be more susceptible to decline in advanced age compared to crystallized cognitive abilities [54]. The speed of processing has been called the hallmark of age-related cognitive decline and it is reasonable to argue that processing speed is more susceptible to age-related decline than other cognitive domains [55, 56].

In this study, the participants had a mean age of 68 years, mostly representing the “younger” elder population. This study therefore reflects early age-related effects on cognition, and also most likely early cardiovascular implications on cognition. A possible explanation, we only found kidney dysfunction to precede decline in processing speed, and no other cognitive domains, could hence be early cardiovascular implications on cognition, affecting the speed of processing prior to other cognitive domains.

The main strength of this longitudinal study is the large number of participants recruited, representing a general population of the (younger) elder population. Another strength is the long follow up time of 6 years, which can be compared to the mean follow up time of 3.2 years, in the review study of Zijlstra et al. [50] mentioned above. Yet another strength is the wide panel of cognitive tests used, covering most of the cognitive domains, and also the small test administrator effects on cognitive performance, since only 1.4–3.5 % of the total variation in test scores was explained by the factor attributed to the test administrator [57].

A limitation in this study is the small number (*n* = 8) of participants with severe kidney dysfunction (eGFR < 30 ml/min/1,73 m^2^), and therefore it was not possible to study the relationship between severe kidney disease and the development of cognitive impairment. Another limitation was the potential loss of individuals with dementia or MCI in this study, due to exclusion of the depressed. Since cognitive impairment can be the result of depression, individuals classified as depressed were excluded. However, depression is also common in the presence of dementia and MCI [58]. Therefore, there is always a risk of excluding the primarily cognitive compromised, when exclusion of individuals with depression is made. However, we performed the calculations including these participants and no clinically relevant differences in the results were found (calculations not shown).

## Conclusions

In conclusion, we found early impairment in renal function to be associated with worsening in speed of processing, but not with incident dementia or MCI, although a fifth of the participants developed cognitive impairment. In our sample, there was a limited representation of participants with severe and end-stage chronic kidney disease, which may have precluded us to observe an association between kidney impairment and dementia and/or MCI. The effect on processing speed could represent early vascular implications on cognition. This suggests that even at moderately impaired kidney function, overview of cardiovascular risk factors is indicated, to prevent future cognitive impairment.

## Data Availability

The data for this study was based on the GÅS dataset. The GÅS dataset is restricted and not publicly available. The data can be obtained by the authors by reasonable request with permission granted from the GÅS study leaders.

## Abbreviations

AD: Alzheimer´s disease
CDR: Clinical dementia rating
CI: Confidence interval
CKD: Chronic kidney disease
CPRS: Comprehensive psychopathological rating scale
CVD: Cardiovascular disease
eGFR: Estimated glomerular filtration rate
GFR: Glomerular filtration rate
GÅS: Good Aging in Skåne
IDMS: Isotope-dilution mass spectrometry
MCI: Mild cognitive impairment
aMCIm: Amnestic MCI multiple domains
aMCIs: Amnestic MCI single domain
naMCIm: Nonamnestic MCI multiple domains
naMCIs: Nonamnestic MCI single domain
MMSE: Mini mental state examination
OR: Odds ratio
SD: Standard deviation
SNAC: Swedish National study on Aging and Care
TMT: Trail making test
